# Body mass index in nursing home residents during the first year after admission

**DOI:** 10.1186/s40795-023-00710-3

**Published:** 2023-03-20

**Authors:** Corinna Vossius, Miguel G. Borda, Bjørn Lichtwarck, Janne Myhre, May Ingvild Volungholen Sollid, Tom Borza, Ingvild Hjorth Feiring, Jūratė Šaltytė Benth, Sverre Bergh

**Affiliations:** 1 The Research Centre for Age-related Functional Decline and Disease, Innlandet Hospital Trust, Ottestad, Norway; 2 Centre for Age-related Medicine, Stavanger University Hospital, Stavanger, Norway; 3 Ageing Institute, Medical School, Pontificia Universidad Javeriana, Bogotá, Colombia; 4 Faculty of Medicine and Health Sciences, Department of Health Sciences, Norwegian University of Science and Technology (NTNU) Gjøvik, Box 191, Gjøvik, 2802 Norway; 5 Health Services Research Unit, Akershus University Hospital, Nordbyhagen, Norway; 6 Institute of Clinical Medicine, University of Oslo, Oslo, Norway; 7 Norwegian National Centre for Ageing and Health, Vestfold Hospital Trust, Tønsberg, Norway

**Keywords:** Nursing home, Nursing home residents, BMI, Weight changes, Malnutrition, Unternutrition, Overweight

## Abstract

**Background:**

Malnutrition - comprising both undernutrition and overweight - has to be addressed in the medical follow-up of older adults due to the negative consequences for the functional state and general health. Still, little is known about the nutritional state of nursing home (NH) residents, especially with respect to weight gain or weight loss after NH admission. Therefore, this study aims to evaluate changes in the body mass index (BMI) during the first year following NH admission, and to explore demographic and clinical characteristics related to BMI changes.

**Methods:**

Data from two prospective studies that recruited participants at NH admission were combined. Demographic and clinical characteristics including the BMI were assessed at baseline and after one year. A linear regression model was estimated to explore the impact of demographic and clinical characteristics on the change in BMI.

**Results:**

The study cohort consisted of 1,044 participants with a mean age of 84.3 years (SD7.6) at baseline; 64.2% were female. At baseline, 33% of the NH residents had severe to moderate undernutrition, while 10% were obese. During the first year of their NH stay, residents with severe to moderate undernutrition had an average increase in BMI of 1.3 kg/m^2^ (SD 2.2; p < 0.001), while weight changes were either very small or not significant in the other BMI groups. Characteristics related to weight gain were younger age and less agitation.

**Conclusion:**

Malnutrition is a common health challenge at NH admission, with one third of NH residents being moderately to severely underweight and 10% being obese. However, during the first year of NH stay, there was a favourable development for underweight NH residents, as they increased their BMI, and 43.6% changed to a higher weight classification, while we observed no changes in the BMI in residents with obesity. As NH residents are in the last phase of their lives, interventions to prevent malnutrition or overweight should be initiated while still home-dwelling, and then continued in the nursing homes.

## Introduction

“*Feed me, feed me, Simon, feed me all night long!”* sings the bloodthirsty Venus flytrap in the Little Shop of Horrors. In contrast to the man-eating plant in the musical, most humans are content with standard food, and their main inclination is that it satisfies their nutritional needs, taste, and eating habits. However, malnutrition is a global challenge, with undernutrition denoting insufficient intake of energy and nutrients to meet an individual’s needs to maintain good health, and overweight as abnormal or excessive fat accumulation that presents a risk to health, and it has to be addressed in the care and medical follow-up of older adults [1, 2]. The Norwegian Directorate of Health estimates that as many as 40% of residents in Norwegian nursing homes (NHs) and persons receiving home care suffer from, or are at risk of, developing undernutrition [3]. Additionally, obesity represents an increasing challenge in nursing home residents [4].

Norwegian NHs are designed for people in need of continuous care and supervision. Mean age at admission is 84 years, and 84% of the long-term residents suffer from dementia [5, 6]. Many residents are no longer capable of identifying and attaining to their basic needs, including nutritional intake, and thus depend on the health care personnel staff to provide adequate nutrition and hydration. A review published by Alzheimer’s Disease International in 2014 described existing research about nutrition in people with dementia [7]. They reported that weight loss might be part of the normal process of aging, and how it is exacerbated in people with dementia, which might lead to several negative consequences. Undernutrition decreases bodily reserves required for stress response and increases the risk of other complications such as infections, falling tendency, fractures, frailty, and sarcopenia [8]. Furthermore, undernutrition is related to longer convalescence periods, increased care needs, and extensive medical expenses [3]. Previous research has shown that undernutrition in NH residents was related to lower functioning in activities of daily living (ADL), having dementia, problems with food intake, higher age, and higher mortality [5, 9–11]. However, reports show that a good nutritional status is not only associated with less disability and lower mortality, but as well with slower cognitive decline in persons suffering from dementia [12–15]. At the same time, a review on studies from the US describes different medical and functional profiles for residents with obesity as compared to normal or underweight residents, younger age at NH admission and a need for more extensive assistance [4].

Still, little is known about the nutritional state of residents at NH admission, especially with respect to weight gain or loss, and demographic and clinical characteristics related to weight changes. Therefore, this study aims to evaluate changes in the body mass index (BMI) during the first year following the NH admission, and to explore demographic and clinical characteristics related to BMI changes.

## Methods

### Settings and participants

We combined data from two prospective clinical studies that recruited participants from 71 NHs in 45 municipalities in Norway at NH admission. The demographic and clinical characteristics from both cohorts were similar. These studies were:

- Resource Use and Disease Course in Dementia - Nursing Home (REDIC-NH) including 696 persons. Inclusion took place between 2012 and 2014 [16].
- Cooperation between The Department of Old Age Psychiatry, Innlandet Hospital Trust, and municipal nursing homes in the Innlandet County (SAM-AKS III). SAM-AKS III is an ongoing study that started in 2014 [17].

Inclusion criteria were: (i) 65 years of age or older in REDIC-NH and 60 years of age or older in SAM-AKS III or (ii) having dementia irrespective of age. (iii) In addition, expected survival should be six weeks or more for REDIC-NH and four weeks or more for SAM-AKS III. Only residents that completed baseline assessment (BL) were included in the studies.

For the present study additional inclusion criteria were: (a) Participants had completed BL within 90 days after NH admission, (b) participants had completed the 12-months follow-up examination (FU12) within one year and 90 days after BL or had died before FU12, and (c) BMI recorded at all assessments.

### Data collection

Data collection was performed by trained healthcare workers at the NH, mainly registered nurses, under supervision of research nurses from the Research Centre for Age-Related Functional Decline and Disease. The research nurses completed a five-day training prior to study start, while the data collectors completed a two-day training. Data were collected through structured interviews with participants and a caregiver [16].

All rating scales and inventories were applied using validated, Norwegian versions. The following demographic and clinical data were collected:

#### Demographic data

Gender, age, and living arrangements before admission to NH, were collected by reviewing patients’ journals.

#### Body mass index (BMI)

relates a person’s weight to the height (BMI = weight/height^2^). The height was assessed at BL by either measuring the resident or by asking the residents or their proxies when the resident was unable to stand erect. The weight was established by weighing the residents at BL and at FU12.

#### The global leadership conversation: addressing malnutrition (GLIM)-criteria

were applied, and the participants were categorized according to their BMI into severe undernutrition (BMI under 18.5 for persons younger than 70 years and under 20 for persons 70 years or older), moderate undernutrition (BMI 18.5 to 20, respectively 20 to 22), normal weight (BMI 20 to 25, respectively 22 to 27) and overweight (BMI over 25, respectively over 27). In addition, participants with a BMI of 30 or higher were classified as obese [2, 18].

#### The clinical dementia rating scale (CDR)

was applied to assess the severity of dementia. The rating scale comprises six items, where the total CDR score is obtained based on an algorithm [19]. For statistical analyses we calculated the CDR-sum of boxes (CDR-SoB) that offers an extended range of values compared to the algorithm-based scoring, and it is calculated by adding the item scores (range 0–18), where higher scores indicate more severe dementia [20].

#### The neuropsychiatric inventory (NPI)

assesses neuropsychiatric symptoms. The instrument contains 12 items and is conducted as an interview with a caregiver. Severity (scored 0–3) is multiplied by frequency (scored 0–4), giving an item score from 0 to 12, where higher scores indicate more severe symptoms [21, 22]. Based on a previous principal component analysis, we created the following sub-syndromes: NPI-Agitation (agitation/aggression, disinhibition, and irritability), NPI-Psychosis (delusions and hallucinations), and NPI-Affective symptoms (depression and anxiety) [16].

#### Physical self-maintenance scale (PSMS)

consists of six items (scored 1–5) and assesses personal activities of daily living (PADL) function. The overall score ranges from 6 to 30, where higher scores indicate higher PADL dependency [23].

#### General medical health rating (GMHR)

rates physical health and was assessed by the health care workers performing the examinations. It consists of one item comprising four categories: excellent, good, moderate, or poor [24].

#### The mobilization-observation-behaviour-intensity-dementia pain scale (MOBID-2)

was applied to assess pain. For this study, we only included the overall proxy rating ranging from 0 to 10, where a higher score indicates more severe pain. [25]

### Ethics

The residents’ capacity to consent to participation in the study was considered by the NH staff, including the NH physician. Written informed consent was obtained by the participants with full capacity to consent, or by next of kin on behalf of the participants in case of reduced capacity to consent. The Regional Ethics Committee for Medical research in South-Eastern Norway approval for the two studies (2011/1378a and 2014/917) includes the analysis for the present study.

### Statistics

Demographic factors and clinical characteristics were presented as means and standard deviations (SDs), or frequencies and percentages. The group differences were assessed by Student’s t-test for continuous variables and χ^2^-test for categorical variables. The analyses comparing participants with severe undernutrition, moderate undernutrition, normal weight, overweight, and obesity according to pre-specified BMI cut-offs were considered exploratory. For some of the exploratory analyses the sub-cohorts of participants with severe and moderate undernutrition were combined. The BMI change between BL and FU12 was assessed by paired samples t-test. A linear regression model was estimated to explore the impact of demographic and clinical characteristics at BL on the change in BMI between BL and FU12. The following characteristics were included: age, gender, living alone before NH admission, GMHR, PSMS, CDR-SoB, the NPI sub-syndromes Agitation, Psychosis and Affective, the overall proxy rating of MOBID-2, and BMI at BL. Due to mathematical coupling and regression to the mean, a straightforward regression analysis between change in BMI and BL BMI might provide biased results. To obtain an accurate estimate of the association between the BMI at BL and the change in BMI, Blomqvist’s method adjusting the estimated coefficient and its standard error for measurement error variance, was applied [26, 27]. Measurement error variance was estimated by employing the BL and FU6 data from the participants of the REDIC study cohort. Due to participants being included from different NH, a cluster effect on NH-level was assessed by intra-class correlations coefficient. As no such effect was found, no adjustment was implemented. Only participants with FU12 assessment and no missing values in covariates were included in the regression analysis. Thus, the sub-cohort of participants not included into the analysis comprised participants deceased before FU12 or with incomplete datasets. Results with p-values below 0.05 were considered statistically significant. The analyses were performed in SPSS v27 and STATA v17.

## Results

**Fig. 1:**
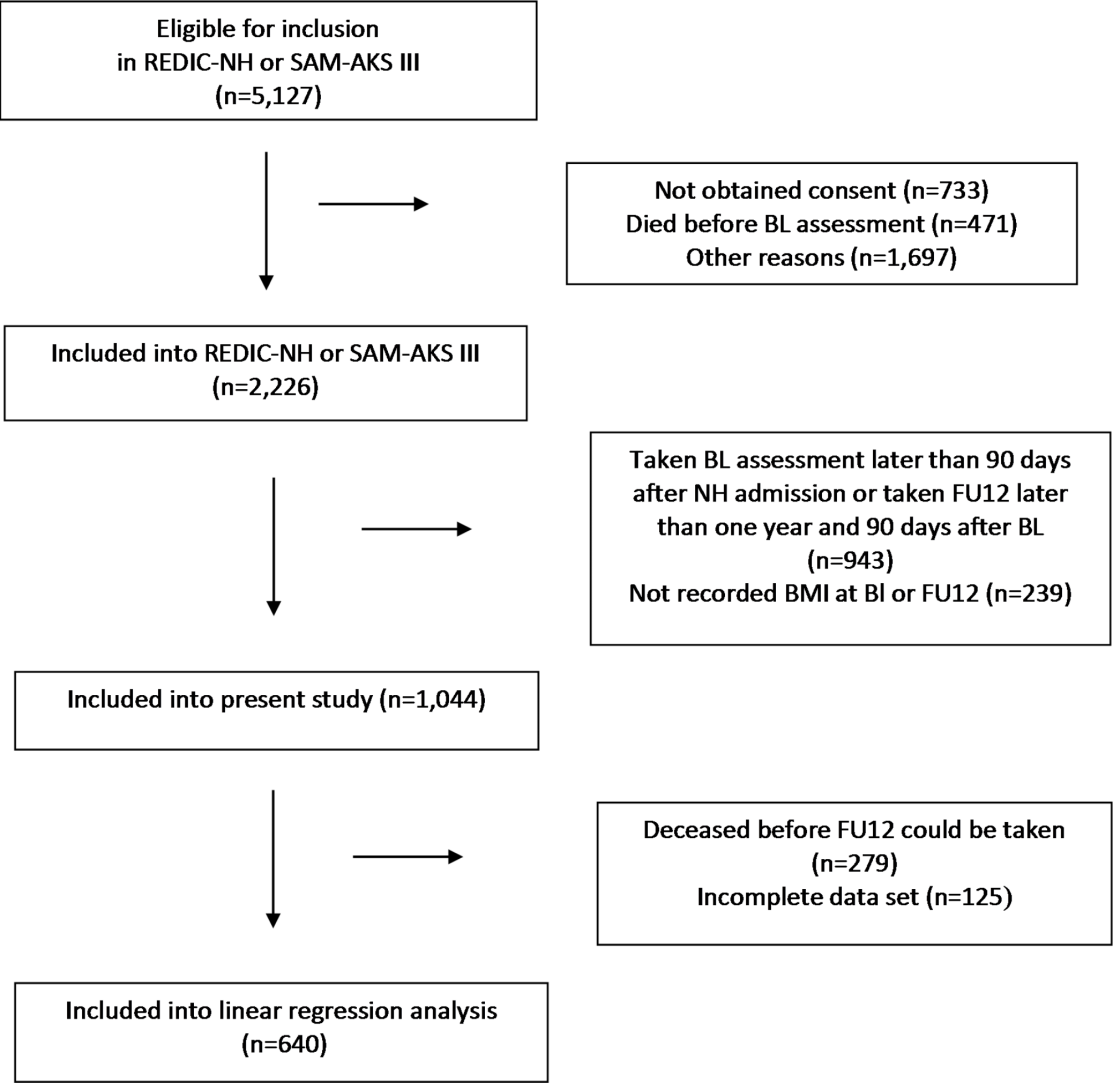
Flow chart of eligible participants and the inclusion process of the study REDIC-NH = Resource Use and Disease Course in Dementia - Nursing Home; SAM-AKS III = Cooperation between The Department of Old Age Psychiatry, Innlandet Hospital Trust, and municipal nursing homes in the Innlandet County. BL = baseline; FU12 = follow-up at 12 months.

As shown in Fig. 1a total of 5,127 persons were eligible for study inclusion, whereof 57% did not participate because they or their next of kin did not consent (14%), the resident died before BL assessment (9%), or other reasons (32%). Those not included were more often male than those included (39 vs. 35%; p < 0.027). A total of 2,226 participants were included into REDIC or SAM-AKS III. Out of these, 932 performed BL assessment within 90 days after NH admission, and FU12 within one year and 90 days after BL. Further, 356 performed BL assessment within 90 days after NH admission, but they died before FU12. Among these 1,283 included participants, BMI was recorded at BL and FU12 in 769 cases, and respectively at BL in 275 participants who deceased before FU12. The study cohort thus consisted of 1,044 participants.

Table 1 shows demographic and clinical characteristics at BL for both included and excluded participants. Except for a higher ADL-dependency, there were no differences between excluded and included participants.

**Table 1:** Demographic and clinical characteristics at BL for included participants and participants excluded from the study, participants included into the regression analysis and those not included into regression analysis, and a comparison of the respective groups

	Included participants	Excluded participants from the study	P Incl. vs. excl. from the study	Participants included into regression analysis	Participants not included into regression analysis	P Incl. vs. not incl. into regression analysis
N	1044	1182		640	404	
Age, mean (SD)	84.3 (7.6)	84.7 (7.7)	0.221	84.0 (7.9)	84.8 (7.2)	0.084
Gender, female (%)	670 (64.2)	754 (63.8)	0.871	408 (63.7)	262 (64.9)	0.718
Living alone before NH admission (%)	716 (68.9)	785 (67.8)	0.592	443 (69.2)	273 (68.4)	0.787
BMI, mean (SD)	24.3 (5.2)	24.9 (4.6)	0.016	24.4 (4.5)	24.0 (6.0)	0.215
GMHR, poor or moderate (%)	486 (49.8)	597 (53.4)	0.087	289 (45.2)	197 (58.8)	**< 0.001**
PSMS, mean (SD)	14.5 (4.5)	15.3 (4.6)	**< 0.001**	13.9 (4.2)	15.5 (4.5)	**< 0.001**
CDR-SoB, mean (SD)	10.2 (4.0)	10.3 (4.5)	0.448	10.1 (3.8)	10.3 (4.4)	0.560
NPI, mean (SD)	14.1 (16.9)	14.3 (17.7)	0.743	12.9 (16.5)	15.9 (17.3)	**0.007**
- NPI-AGI - NPI-PSY - NPI-AFF	5.4 (8.3) 1.8 (3.9) 4.1 (6.5)	5.2 (8.4) 1.8 (3.9) 4.6 (6.7)	0.520 0.672 0.137	3.7 (6.1) 1.7 (3.8) 5.0 (8.3)	4.9 (7.0) 1.8 (4.0) 6.0 (8.3)	**0.008** 0.827 0.069
MOBID-2, mean (SD)	2.1 (2.1)	2.3 (2.2)	0.119	1.9 (2.1)	2.0 (2.2)	0.292

BL = baseline; incl.=included; excl.=excluded; SD = standard deviation; GMHR = General medical health rating; BMI = Body mass index; PSMS = Physical self-maintenance scale; NPI = Neuropsychiatric inventory; NPI-AGI = NPI sub-syndromes agitation/aggression, disinhibition and irritability, NPI-PSY = NPI sub-syndromes delusions and hallucinations; NPI-AFF = NPI sub-syndromes depression and anxiety; CDR-SoB = Clinical dementia rating scale – sum of boxes; MOBID-2 = The Mobilization-Observation-Behaviour-Intensity-Dementia Pain Scale, overall proxy rating.

Table 2 shows demographic and clinical characteristics at BL stratified according to weight classification. About one third of the participants had severe or moderate undernutrition, and about 10% were obese. Explorative analyses showed that participants with severe to moderate undernutrition at NH admission were older (p = 0.001), had more neuropsychiatric symptoms (p = 0.019), were living more often alone before NH admission, and had a higher risk to die before FU12 (p = 0.029) than participants with normal weight or overweight. Obesity was related to lower age at NH admission (p = 0.023), lower general health state (p < 0.001), and lower degree of cognitive impairment (p = 0.012) when compared to the other weight groups. Both residents with severe undernutrition and those with obesity suffered from more pain (p = 0.012 and p = 0.002, respectively) when compared to the other weight groups. On average, the BMI increase was 0.6 kg/m^2^ (SD 2.5, p < 0.001) between BL and FU12. Persons with severe to moderate undernutrition had the highest increase in BMI, with 1.3 kg/m^2^ (SD 2.2; p < 0.001) and 43.6% of residents in these weight classifications changed to a higher weight classification during their first year of NH stay.

**Table 2:** Demographic and clinical characteristics at BL, stratified according to weight classification, and BMI changes and changes in weight classification between BL and FU12.

	All	Severe under-nutrition	Moderate under-nutrition	Normal weight	Overweight	Obesity
N	1044	169 (16.2)	178 (17.0)	453 (43.2)	139 (13.3)	107 (10.2)
Age, mean (SD)	84.3 (7.6)	85.6 (7.0)	85.1 (7.0)	84.3 (7.6)	82.7 (8.4)	82.3 (7.8)
Gender, female (%)	670 (64.2)	127 (75.1)	118 (66.3)	266 (59.0)	85 (61.2)	74 (69.2)
Living alone before NH admission (%)	716 (68.9)	128 (75.7)	125 (70.2)	301 (67.2)	69 (64.0)	73 (68.9)
BMI, mean (SD)	24.3 (5.2)	17.9 (1.6)	21.0 (0.6)	24.4 (1.5)	28.2 (1.0)	34.2 (6.8)
GMHR, poor or moderate	486 (49.8)	84 (49.7)	79 (47.3)	193 (45.7)	63 (48.5)	67 (67.0)
PSMS, mean (SD)	14.5 (4.5)	14.9 (4.7)	14.5 (4.6)	14.2 (4.2)	14.7 (4.5)	14.7 (3.9)
CDR-SoB, mean (SD)	10.2 (4.0)	10.7 (4.1)	10.0 (4.1)	10.2 (3.9)	10.4 (3.8)	9.2 (4.1)
NPI, mean (SD)	14.1 (16.9)	15.5 (16.5)	16.1 (17.9)	13.9 (17.6)	11.6 (14.6)	12.1 (15.0)
- NPI-AGI - NPI-PSY - NPI-AFF	4.1 (6.5) 1.8 (3.9) 5.4 (8.3)	4.0 (6.0) 1.9 (4.4) 5.6 (8.6)	4.9 (7.1) 2.1 (4.2) 6.2 (9.5)	4.3 (6.8) 1.7 (3.8) 5.4 (8.5)	3.6 (5.3) 1.4 (3.1) 4.4 (6.4)	3.3 (5.6) 1.6 (3.6) 4.6 (7.1)
MOBID-2, mean (SD)	2.1 (2.1)	2.4 (2.2)	2.0 (2.2)	1.7 (2.0)	1.7 (1.7)	2.5 (2.4)
Deceased before FU12	275 (26.3)	58 (34.3)	48 (27.0)	115 (25.5)	33 (23.7)	21 (19.6)
BMI change between BL and FU12 (95%CI)	0.6 (0.4–0.8)	1.6 (1.2–2.1)	1.0 (0.6–1.4)	0.4 (0.1–0.7)	0.4 (0–0.8)	-0.3 (-1.0–0.4)
Changes in weight classification between BL and FU12 (%) - Change to a higher group - Change to a lower group	221 (28.7) 69 (9.0)	44 (39.6) -	61 (46.2) 17 (12.9)	79 (23.5) 20 (6.0)	37 (31.7) 18 (17.0)	- 14 (16.3)

BL = baseline; FU12 = follow-up at 12 months; SD = standard deviation; GMHR = General medical health rating; BMI = Body mass index; PSMS = Physical self-maintenance scale; NPI = Neuropsychiatric inventory; NPI-AGI = NPI sub-syndromes agitation/aggression, disinhibition and irritability, NPI-PSY = NPI sub-syndromes delusions and hallucinations; NPI-AFF = NPI sub-syndromes depression and anxiety; CDR-SoB = Clinical dementia rating scale – sum of boxes; MOBID-2 = The Mobilization-Observation-Behaviour-Intensity-Dementia Pain Scale, overall proxy rating.

Table 3 presents the results of the linear regression model assessing the association between the change in BMI between BL and FU12 and demographic and clinical characteristics at BL. In both the bivariate and multiple models, the BMI at BL is significantly negatively correlated to the change in BMI from BL to FU12. However, after Blomqvist’s adjustment, the association between BMI at BL and the change in BMI is no longer significant. According to the Blomqvist-adjusted multiple model, there would be less weight gain and respectively a higher weight loss with higher age and with more symptoms of agitation.

**Table 3:** Results of the linear regression model for association between the change in BMI between BL and FU12 and demographic and clinical characteristics at BL.

Covariate	Bivariate models		Multiple model	
	Regr.coeff. (95% CI)	p-value	Regr.coeff. (95% CI)	p-value
BMI BL – unadjusted* BMI BL – adjusted** Age Gender, female Living alone, yes GMHR, poor/moderate PSMS CDR-SoB NPI-AGI NPI-PSY NPI-AFF MOBID-2	-0.12 (-0.16; -0.07) -0.02 (-0.07; 0.04) 0.001 (-0.02; 0.03) 0.45 (0.05; 0.84) 0.57 (0.16; 0.98) -0.38 (-0.77; 0.001) -0.06 (-0.10; -0.01) -0.07 (-0.12; -0.01) -0.04 (-0.07; -0.01) -0.03 (-0.08; 0.02) -0.03 (-0.05; -0.01) -0.05 (-0.14; 0.05)	**< 0.001** 0.498 0.949 **0.028** **0.007** 0.051 **0.014** **0.012** **0.009** 0.241 **0.007** 0.310	-0.12 (-0.16; -0.08) -0.02 (-0.08; 0.04) -0.03 (-0.05; -0.002) 0.30 (-0.10; 0.70) 0.37 (-0.06; 0.80) -0.18 (-0.57; 0.21) -0.01 (-0.06; 0.04) -0.04 (-0.10; 0.02) -0.04 (-0.08; -0.00004) 0.01 (-0.05; 0.07) -0.02 (-0.05; 0.007) -0.05 (-0.14; 0.05)	**< 0.001** 0.468 **0.033** 0.143 0.090 0.361 0.634 0.143 **0.0498** 0.772 0.154 0.349

Regr.coeff.=Regression coefficient; CI = Confidence interval; BL = baseline; GMHR = General medical health rating; BMI = Body mass index; PSMS = Physical self-maintenance scale; NPI = Neuropsychiatric inventory; NPI-AGI = NPI sub-syndromes agitation/aggression, disinhibition and irritability, NPI-PSY = NPI sub-syndromes delusions and hallucinations; NPI-AFF = NPI sub-syndromes depression and anxiety; CDR-SoB = Clinical dementia rating scale – sum of boxes; MOBID-2 = The Mobilization-Observation-Behaviour-Intensity-Dementia Pain Scale, overall proxy rating.

*Unadjusted for measurement error variance; ** adjusted for measurement error variance by Blomqvist’s method.

## Discussion

At NH admission we observed that about 33% of the NH residents had severe to moderate undernutrition, while 10% were obese. Residents with undernutrition were older, living more often alone before NH admission, had more symptoms of agitation, and a higher mortality rate. Residents with obesity where younger, experienced more pain, had a lower general health state and less cognitive impairment. During the first year of their NH stay, persons with severe to moderate undernutrition had an average increase in BMI of 1.3 kg/m^2^, while weight changes were either very small or not significant in the other BMI groups. Weight gain was associated with younger age and less agitation.

Our findings regarding undernutrition are in line with previous research that reports a prevalence of undernutrition of 30% in Swedish NHs [28]. The findings also reflect well the nutrition states in home-dwelling persons with dementia in Norway, where 29% were found to be underweight at the time of the diagnosis of dementia [11]. Underweight residents had a higher mortality rate during their first year of NH stay, and these results are also in accordance with previous research, possibly indicating that weight loss is part of the natural process of dying of age [5, 29]. Residents with undernutrition represented the sub-cohort with the highest weight gain and 43.6 changed into a higher weight classification group during their first year of NH stay, indicating that the NHs succeeded in providing adequate nourishment. Unfortunately, we lack data whether the follow-up of the residents involved screening for malnutrition and creating customized food plans, as required by the national guidelines, or if the observed weight gain was a more coincidental result of regular meals and the general availability of food or an indicator of general thriving. In addition, we observed that 12.9% of persons with moderate undernutrition at BL were classified as severely undernourished at FU12. These cases would especially warrant a clarification of the reasons for weight loss and individualized interventions.

However, our findings contradict quite frequent reports in Norwegian media about insufficient nutrition of NH residents and patients supposedly “starving to death” [30–32]. Rather than created at the NH, malnutrition seems to be a problem that arises prior to NH admission. In the exploratory analyses we found that residents with malnutrition at BL more often were living alone while still home-dwelling, indicating that this group might be especially vulnerable for malnutrition. The linear regression model identified two risk factors for weight loss: age and agitation. While age is a factor beyond intervention, agitation might be approached by trying to identify underlying and treatable health issues like for example pain or urinary tract infections, or by targeted interventions to decrease agitation itself.

In residents with overweight, we see that almost one third is classified as obese after one year of NH stay, increasing the share of residents with obesity from 10 to 14%. Additionally, we observed that residents who suffered from obesity at BL, were the group with no significant change in BMI. A previous study reported a prevalence for obesity of 18% in a French NH population, with no weight change during the two-month observation period, despite dietary regimes. As in our study, pain was more frequently reported in residents with obesity as compared to the other BMI groups [33]. As described above, obesity represents a risk factor for NH admission, due to the general health risk of overweight and due to increased functional decline, resulting in both a higher rate of NH admissions and at a younger age. However, there has not been observed increased NH mortality, resulting in a longer NH stay for this patient group [4, 29]. As obesity is an increasing public health problem, also in Norway, this might enhance the foreseen need for more NH beds in the decades to come [34, 35]. This raises the question, whether NHs should implement interventions that aim at weight control. According to the current ESPEN guidelines on nutrition and hydration in geriatrics, interventions to lose body weight are not recommended for older people with overweight [36]. Weight loss interventions are only considered beneficial when combined with exercise to retain muscle mass. However, randomized controlled trials with nursing home residents are lacking and most studies include people between 65 and 70 years without severe functional limitations.

### Strengths and limitations of the study

We followed a cohort of 1,044 participants in a longitudinal study from NH admission and during the first year of their long-term NH stay, with clinical examinations at BL and after 12 months. For clinical, prospective studies in the NH setting cohorts of about a thousand participants are scarce. The study cohort consisted of NH residents from both urban and rural municipalities. High quality of the data collection was secured by a standardized interview carried out by healthcare workers with adequate training under the supervision of research nurses. Furthermore, the Norwegian health and social system provides a rather homogenous environment for health service research as there are hardly any private sector healthcare providers on the market. The decision of NH admission is made by care workers in the municipality administration, mainly based on the patients’ functional status and with comparable thresholds for admission.

Still, the main weakness of this study is that our sample might not be representative of the general NH population in Norway, as less than half of all eligible residents participated in REDIC-NH and SAM-AKS III, and as the stricter inclusion criteria of the present study resulted in an additional attrition of study participants. Further, the BMI was only assessed at two time points one year apart. Thus, we know nothing about the changes in BMI of the deceased participants. The nutritional state was solely assessed by BMI, as we lacked information about comorbidities or possible problems with food intake. The BMI does not always represent the full nutritional status of an older person, as it does not necessarily reflect changes in a person’s percentage of muscle mass and body fat. This might have led to an underestimation of malnutrition.

## Conclusion

Malnutrition is a common health challenge at NH admission, with one third of NH residents being moderately to severely underweight and 10% being obese. However, during the first year of NH stay, there was a favourable development for underweight NH residents, as they increased their BMI, and 43.6% changed to a higher weight classification, while we observed no changes in the BMI in residents with obesity. As NH residents are in the last phase of their lives, interventions to prevent malnutrition or overweight should be initiated while still home-dwelling, and then continued in the nursing homes.

## Data Availability

The datasets generated and analysed during the current study are not publicly available as public availability was not consented to by the study participants and not approved by the Ethics Committee. Data is available from the corresponding author on reasonable request.
